# How allergenic are hypoallergenic formulae in patients with severe cow’s milk protein allergy?

**DOI:** 10.1186/2045-7022-1-S1-P12

**Published:** 2011-08-12

**Authors:** Angela Claver, Javier Boné, Isabel Guallar

**Affiliations:** 1 Hospital Universitario Miguel Servet, Pediatric Allergy, Zaragoza, Spain

## Background

In patients diagnosed of severe IgE- mediated cow's milk protein allergy (CMPA), skin prick tests (SPTs) using special milk formulae, can provide useful information, not only on the clinical course and severity of each case, but also on the tolerance and allergenicity of the tested substitute milks.

## Materials and Methods

In the last 5 years (2005-2009) 7-8 patients (9-17 years) diagnosed with severe CMPA were evaluated with SPTs and cow's milk specific -IgE in vitro determination (casein sIgE levels varied between 126 and 884 KU/L). We performed the SPTs for 17 different special formulae: 1 conventional infant formula (CMF), 3 partially hydrolysed whey/casein ones (pHF-W), 4 extensively hydrolysed casein formulae (eHF-C), 2 extensively hydrolysed whey formulae (eHF-W), 2 soya formulae (SF), 1 soya/collagen hidrolyzed mixture (HF-S) and 1 amino acid-derived formula (AaF) .In 2009, we added 3 extensively hydrolysed formulae which contained lactose (2 eHF-W and one pHF-C).

## Results

All the patients presented positive SPTs (wheal diameter >3mm ) for CMF, pHF-W, eHF-C and eHF-W with the exception of Alfaré Nestlé ® and Althéra Nestlé ® (eHP-W), which were negative in all cases. SPTs with SF, HF-S and AaF were negative in all the children excluding the 2008 results (positive results in the whole group for a SF because of a batch contamination that was later comfirmed).

## Conclusions

High degree of sensitization in our patients , provides an "in vivo" evaluation of the substitute milk formulas (determining their residual allergenicity and the possibility for tolerance in children with CMPA) Also, minimum contaminations or changes in the formula composition are detected with great precision. We would like to highlight the results obtained with Alfaré ® and Althéra ® : despite being eHF-W behaved like AaF in the SPTs.

